# Manage at work: a randomized, controlled trial of a self-management group intervention to overcome workplace challenges associated with chronic physical health conditions

**DOI:** 10.1186/1471-2458-14-515

**Published:** 2014-05-28

**Authors:** William S Shaw, Elyssa Besen, Glenn Pransky, Cécile RL Boot, Michael K Nicholas, Robert K McLellan, Torill H Tveito

**Affiliations:** 1 Liberty Mutual Research Institute for Safety, 71 Frankland Rd., Hopkinton, MA 01748, USA; 2 University of Massachusetts Medical School, 55 N. Lake Ave., Worcester, MA 01655, USA; 3 VU University Medical Center, EMGO Institute for Health and Care Research, Department of Public and Occupational Health, Van der Boechorststraat 7, 1081 BT Amsterdam, The Netherlands; 4 University of Sydney at Royal North Shore Hospital, St. Leonards, NSW 2065, Australia; 5 Geisel School of Medicine at Dartmouth, 1 Rope Ferry Drive, Hanover, NH 03755, USA; 6 Uni Health, Uni Research, Christiesgate 13, N-5015 Bergen, Norway; 7 Clinic Physical Medicine and Rehabilitation, Vestfold Hospital Trust, Stavern 3290, Norway

**Keywords:** Chronic health conditions, Workplace, Coping, Pain management, RCT, Presenteeism

## Abstract

**Background:**

The percentage of older and chronically ill workers is increasing rapidly in the US and in many other countries, but few interventions are available to help employees overcome the workplace challenges of chronic pain and other physical health conditions. While most workers are eligible for job accommodation and disability compensation benefits, other workplace strategies might improve individual-level coping and problem solving to prevent work disability. In this study, we hypothesize that an employer-sponsored group intervention program employing self-management principles may improve worker engagement and reduce functional limitation associated with chronic disorders.

**Methods:**

In a randomized controlled trial (RCT), workers participating in an employer-sponsored self-management group intervention will be compared with a no-treatment (wait list) control condition. Volunteer employees (n = 300) will be recruited from five participating employers and randomly assigned to intervention or control. Participants in the intervention arm will attend facilitated group workshop sessions at work (10 hours total) to explore methods for improving comfort, adjusting work habits, communicating needs effectively, applying systematic problem solving, and dealing with negative thoughts and emotions about work. Work engagement and work limitation are the principal outcomes. Secondary outcomes include fatigue, job satisfaction, self-efficacy, turnover intention, sickness absence, and health care utilization. Measurements will be taken at baseline, 6-, and 12-month follow-up. A process evaluation will be performed alongside the randomized trial.

**Discussion:**

This study will be most relevant for organizations and occupational settings where some degree of job flexibility, leeway, and decision-making autonomy can be afforded to affected workers. The study design will provide initial assessment of a novel workplace approach and to understand factors affecting its feasibility and effectiveness.

**Trial registration:**

Clinicaltrials.gov: NCT01978392 (Issued November 6, 2013)

## Background

One dramatic workforce trend in the US and elsewhere is the advancing median age of workers and the growing prevalence of chronic health conditions that contribute to workplace pain, fatigue, task limitations, and reduced productivity. Approximately 40 percent of working U.S. adults report persistent or recurrent musculoskeletal pain conditions or other chronic physical health conditions that limit their ability to work in measures of point prevalence [1,2]. Because of aging trends in the workforce, the prevalence of chronic diseases will increase in coming years, especially musculoskeletal conditions [3]. Even among younger workers, the prevalence of obesity and chronic health symptoms has increased [4,5]. The most disabling chronic conditions among working age adults are low back pain, arthritis, migraine headaches, depression, diabetes, heart disease, and asthma [6-8]. For ill workers, the ability to continue working represents a serious quality-of-life issue with significant financial and lifestyle implications [9]. This problem also increases employer costs through reduced productivity, high turnover rates, absenteeism, and health care expense [10-13].

By definition, chronic health conditions represent recurrent or long-lasting problems that never completely remit; thus, maintaining daily functioning and quality-of-life is an important aim of intervention. Despite having functional limitations, the majority of working-age adults with chronic conditions desire gainful employment, and most are able to accomplish this without the need for formal job accommodations or physician-ordered restrictions [14-16]. Qualitative interviews with workers suggest this is possible by leveraging available job leeway and flexibility, by careful planning and decision-making with regard to work, by obtaining job assistance and social support in and out of work, and by communicating needs effectively and judiciously with peers and supervisors [17,18]. What has not been studied is whether specific employer-supported organizational or educational interventions might help workers to build on these coping resources to improve workplace function and well-being.

While employer accommodation and nondiscriminatory policies and practices are critical to prevent unnecessary cases of work disability, another possible strategy is to improve workplace coping and function by offering coaching and support to affected workers. In this study, we hypothesize that interventions employing principles of pain and illness self-management (SM) may be effective when adapted to the workplace context. SM interventions apply peer support and psycho-educational techniques borrowed from cognitive-behavioral therapy to enhance coping skills and provide individualized plans for problem solving and dealing with temporary setbacks [19,20]. The self-management approach attempts to redefine health symptoms and functional challenges as subject to personal control and mastery through the encouragement of an active, problem-solving perspective [21,22]. Identifying and modifying negative cognitions is another important instructional element [20,23]. SM interventions have consistently shown reductions in pain, fatigue, functional limitations, and distress in clinical trials [21,24-33], but these prior studies have not focused on workplace problems [29].

While many employers offer return-to-work assistance and temporary job modifications after a prolonged period of sickness absence, there are few employer policies or programs designed to address the day-to-day problems of workers with chronic physical health conditions. Therefore, the aim of this study will be to test the effectiveness of an employer-sponsored self-management group intervention when offered to workers with chronic physical health conditions. We hypothesized that such a program would show greater improvements in work engagement and a reduction in work limitations compared to a wait-list control condition.

## Methods

### Study context

In the US, employers are required to provide reasonable accommodation for employees with disabling health conditions, and a job position must be held open for a sick worker from 3 months to several years, depending on the state of jurisdiction and whether the illness is deemed work-related. Intermittent health problems, with recurring flare-ups and/or short, periodic absences from work represent a special challenge for employers, and workers with chronic conditions are at greater risk for job loss in the US than in many other countries. While many large employers in the US offer employees programs in health promotion, health risk appraisal, and wellness coaching, few of these programs offer advice for managing chronic health conditions while also keeping up with work demands. Disability and insurance benefits for non-work related conditions can vary and depend on employee-paid private insurance coverage.

### Study design

**Figure 1:**
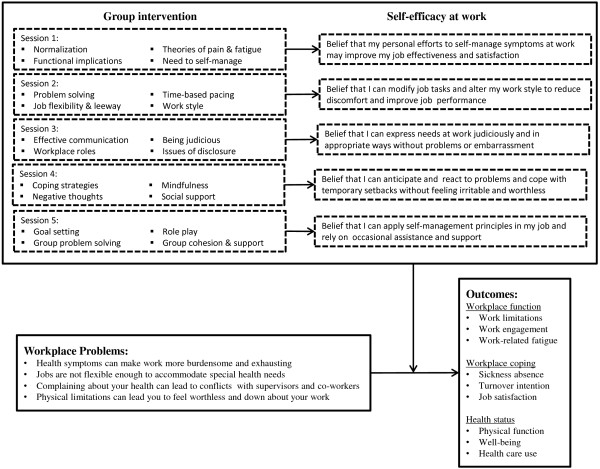
The conceptual framework for the intervention focusing on self-efficacy principles.

The proposed study design is a randomized, controlled trial (RCT) of an employer-sponsored psycho-educational group intervention program designed to improve workplace functioning among workers with chronic physical health symptoms (Clinical Trials Registry # NCT01978392). The study methodology involves recruiting employees with chronic conditions from five worksites, randomizing them to participate in a group intervention program or to a wait-list control arm, and assessing changes in baseline worker engagement and work limitations at 6- and 12-month follow-up. We hypothesize that group participation will improve health and disability outcomes by improving self-efficacy beliefs in several work-related domains (see Figure 1). A process evaluation will be conducted alongside the RCT. The study has been reviewed and approved by the Dartmouth College Committee for the Protection of Human Subjects (#24084).

### Study population

The study population is workers who have at least one chronic physical health condition (>6 months) and are interested in exploring ways to deal effectively with health-related challenges associated with body aches, discomfort, and fatigue on the job. The recruitment procedure will include general workforce announcements (email, posters, flyers, employee newsletter) as well as individual notices to workers accessing wellness services or interacting with on-site employee healthcare providers. All inquiries about the project will be forwarded to a local project coordinator who will answer questions, obtain informed consent, administer the baseline survey, and schedule employees for workshop meetings.

### Inclusion criteria

Participants will be full-time workers (>20 hours per week) of age 18 years or older who self-report at least one chronic physical health condition (>6 months). All participants must read and speak in English. To prevent any unnecessary health disclosure, interested employees will not be required to divulge medical information about diagnoses and symptoms to the on-site project coordinator in order to qualify for the study; however, this background health information will be collected as part of the baseline survey after recruitment.

### Exclusion criteria

There are no diagnostic exclusions, but workers with an impending retirement or major job change (next 12 months) will be excluded, as will those who report no availability to attend group workshops before work, after work, or during lunch breaks. Workers who are currently out of work on a disability leave (>2 weeks) and workers unable to read and communicate in English will not be eligible for the study.

### Randomization and blinding

Randomization will occur at the individual level after workers provide written informed consent and complete the baseline survey. As recruitment of workers will occur over a period of time, a computer-generated block randomization schedule (with equal sized groups for every 20 participants) will be maintained by the project statistician and used to assign individuals to the intervention or control conditions, and separate randomization schedules will be maintained for each of the five participating employers. Allocation will be concealed and the randomization schedules will be kept confidential and secured by the project statistician, so group assignment will not be anticipated by those involved in patient recruitment. The research associate responsible for collecting and compiling follow-up survey data and the researchers performing data analyses will be kept blind to group assignment. The nature of the psycho-educational group intervention will make it impossible to blind study participants or group facilitators from randomization results.

### Group intervention strategy

Participants randomized to the intervention arm will be assigned to participate in five 2-hour group workshop sessions (or ten 1-hour sessions, if necessary to meet scheduling constraints) led by a specially trained facilitator (licensed psychologist or clinical social worker) and provided over a span of approximately 2–3 months. The sessions will be offered at the work site but not during working hours. The content of the intervention was developed from qualitative studies [30] and from a review of existing self-management intervention elements [29], then piloted and revised according to participant feedback. The intervention incorporates standard elements of existing evidence-based pain and illness self-management efforts but tailors key messages and discussion elements to workplace problems most relevant to workers with chronic physical health conditions. The theoretical basis for the intervention (Figure 1) is Bandura’s Self-Efficacy component of Social Cognitive Theory, which suggests that “the self-assurance with which people approach and manage difficult tasks determines whether they make good or poor use of their capabilities” (p. 35) [34]. Each session is focused on different self-management strategies, with each session containing a mix of facilitator presentation, group discussion, case illustrations, role play, completion of in-session self-assessments and activities, and brief homework assignments. Participants are encouraged to support each other through communications outside of the scheduled meetings, though this interaction is voluntary. Approximately equal time is allocated to the topics of improving comfort, modifying work, communicating effectively, applying systematic problem-solving strategies, and dealing with negative thoughts and emotions (see Table 1).

**Table 1:** Goals and key points for group sessions of the self-management intervention

	**Title**	**Session goals**	**Key points**
Session I	Self-management principles for coping with chronic pain and fatigue	Provide introductions; establish rapport; provide a scientific and philosophical rationale; differentiate self-management from medical management; recognize workplace challenges and constraints; set the general scope and agenda for future sessions	• Chronic physical health conditions are common.
			• Coping at work can require special skills.
			• Thoughts, emotions, and behavior play a part.
			• You are your own best expert and advocate.
			• Coaching and social support can help.
			• Mindfulness and problem solving can help.
Session II	Job modification, pacing, and problem solving	Understand problematic job tasks; apply principles of mindfulness to workplace activity and discomfort; identify potential sources of leeway and flexibility; brainstorm possible opportunities for modifying work organization and work style; apply 6-step problem solving process.	• Some job tasks are more difficult than others.
			• Some job tasks may be adjustable.
			• Leeway and flexibility can be an advantage.
			• Manage your work to the extent possible.
			• Identify functional challenges and constraints.
			• Apply systematic problem solving steps.
Session III	Communicating about health problems at work	Explore different reasons for communicating with others at work about pain and fatigue; identify effective communication strategies; discuss personal choices for disclosure, recognize unique aspects of workplace rules and working roles that affect communication.	• Pain and fatigue can interfere with communication.
			• A need may arise to discuss health at work.
			• Make disclosure decisions judiciously.
			• Communicating about pain can impact others.
			• Understand reasons and context for talking about pain.
			• When needed, direct assertive communication is best.
Session IV	Keeping a positive outlook and adopting realistic goals	Acknowledge the negative effect of pain and fatigue on emotions; recognize negative automatic thoughts about health and work; identify ways to accept more moderate and rational expectations about work performance; suggest coping strategies for dealing with temporary setbacks and discouragement.	• Pain and fatigue can trigger negative automatic thoughts
			• Negative self-talk can impair your job performance.
			• Negative self-talk can make your symptoms worse.
			• Accepting more realistic job expectations can help.
			• Be mindful of pain, not overwhelmed by pain.
			• Have a plan for coping with stress & temporary setbacks.
Session V	Putting it all together: Taking care of yourself at work	Integrate principles of workplace coping, problem solving, job task alteration, and workplace communication through hypothetical case scenarios; foster individual goal setting; summary and closure.	• Health-related challenges are complex.
			• Problem solving can improve work style and pacing.
			• Communicating effectively can improve support at work
			• Keeping a positive, rationale attitude is critical.
			• Be mindful of symptoms while working.

### Non-treatment control strategy

Participants randomized to the control arm will receive no intervention during the 12-month period of study participation. However, after completing their 12-month follow-up, participants in the control group will be invited to attend a full-day Saturday workshop. The intent of the full-day workshop will be to provide the same self-management information as in the intervention arm, but on a delayed basis and in a more condensed format. The full-day workshop will be led by the same trained facilitators as the intervention group and participants will receive all of the same materials.

### Use of co-interventions

As part of each assessment, participants will be asked to report the frequency and type of medical and rehabilitation services used over the previous 6 months at both follow-up assessments.

### Primary outcome measure

#### Work limitation

The Work Limitations Questionnaire (WLQ) [35] is a 25-item self-report questionnaire that assesses the degree to which working individuals are experiencing limitations on-the-job due to their health problems and health-related productivity loss. The WLQ items ask respondents to rate their level of difficulty or ability to perform specific job demands. Items are grouped into 4 scales: (1) time management, (2) physical demands, (3) mental-interpersonal demands, and (4) output demands. The individual scales have shown good internal consistency (Cronbach’s alpha > .70) and they have been validated against other health and disability constructs [36]. Most importantly for the current study, the WLQ has been correlated with objectively-measured employee-level work productivity, and scores on the WLQ can be translated into a single Productivity Index score that indicates the percentage difference in output from a healthy (not limited) benchmark population [37]. Recently, the WLQ has also been shown sensitive to the effects of intervention [38].

### Secondary outcome measures

#### Work engagement

The Utrecht Work Engagement Scale (UWES) is a 17-item self-report questionnaire that was designed to measure the degree to which employees have a sense of energetic and effective connection with their work activities (energy, involvement) and see themselves as able to deal with the demands of their job (professional efficacy) [39]. We will use the shortened 9-item version which has been previously validated [40]. Respondents rate their level of agreement with stated feelings about work on a 7-point likert scale from “never” to “always”. Originally conceptualized as the opposite of job burnout, work engagement has begun to receive a high level of attention in organizational research, and work engagement questions are now commonplace in large-scale employee opinion surveys. Work engagement has been defined as a positive, fulfilling, work-related state of mind that is characterized by vigor, dedication, and absorption. We chose this outcome measure because our prior qualitative work showed that disengagement from work was a greater concern to workers with health issues than any lapse in productivity.

#### Work fatigue

The Occupational Fatigue Exhaustion Recovery Scale (OFER) is a 20-item self-report questionnaire that assesses the degree to which job activities produce acute fatigue, deplete available energy, and reduce the ability to engage in pleasurable activities outside of work [41]. Respondents rate their level of agreement on a 7-point likert scale from “completely disagree” to “completely agree”. The measure shows good test-retest reliability and confirmatory factor analyses have shown good support for its construct validity [42]. We chose this outcome measure based on our prior qualitative work, which revealed a high level of exhaustion and inactivity by individuals with chronic pain after returning home from work.

#### Turnover intention

A 4-item scale developed by Kelloway and colleagues [43] will be used to assess turnover intentions. The four questions are: “I am thinking about leaving this organization”, “I am planning to look for a new job”, “I intend to ask people about new job opportunities,” and “I don’t plan to be in this organization much longer”. Each item is rated on a 5-point scale from “strongly disagree” to “strongly agree”. Internal consistency of the measure is high (Cronbach’s alpha of .92), and studies have shown this construct to be an important outcome of job stress and job strain and a likely precursor to resignation [44]. We chose this outcome measure because of the potential indirect cost to employers for hiring and retraining if workers with chronic health conditions resign when no longer able to cope with job demands.

#### Job satisfaction

A single item will be used to assess job satisfaction. The item is “Please indicate how you would rate your current work situation” on a scale ranging from 1 “worst to 10 “best”. Single item measures of job satisfaction have been argued to have better face validity and to be better able to capture changes in job satisfaction [45,46]. Single item measures of job satisfaction have been found to highly correlate with full scale measures [47].

#### Self-efficacy

A unique self-efficacy measure was developed for the project to be closely aligned with the content of the workshop program. Ten items were adapted from the Pain Self-efficacy Questionnaire [48] and ten items were taken from the Return-To-Work Self-Efficacy (RTWSE-19) scale [49], with the overall goal of assessing worker confidence with respect to: (1) symptom management; (2) job modification; (3) communication; (4) emotional coping; and (5) obtaining needed support and assistance.

#### Sickness absence

Participants will be asked to recall the number of days in the past 6 months that they were absent from work because of their health. Self-report has been shown to be a viable and reasonably accurate method for assessing sickness absence days in studies of employee health [50].

#### Healthcare utilization

Participants will be asked to recall the number and types of health care visits over the prior 6 months using a standardized set of reporting options. Self-report has been shown to be a viable and reasonably accurate method for assessing health care utilization rates in studies of employee health [50] and among individuals with chronic conditions [51].

#### Work environment

The Areas of Worklife Survey (AWS) [52,53] will be used to assess basic perceptions of workload, organizational support, and psychosocial work environment. The 28-item AWS assesses the workplace with regard to six workplace dimensions: (1) workload; (2) control; (3) reward; (4) community; (5) fairness; and (6) values. Respondents rate their level of agreement on a 5-point scale from “strongly disagree” to “strongly agree”.

### Covariates and potential confounders

#### General health status

The SF-12 Health Survey [54] will be used to assess general health status at the baseline assessment. This is the most well validated and frequently used measure of generic health status in health research. The SF-12 provides a single metric of health function regardless of diagnostic categories or illness classifications. For the current study, this will provide a single, uniform measure for characterizing illness severity across participants with varying health conditions. This measure contains subscales for both mental health and physical health.

#### Flexibility of work

The Job Leeway Scale (JLS) is a new 18-item measure developed by the authors (THT, WSS) from the qualitative results of focus groups [30]. This measure will be used to assess the extent to which study participants feel their jobs offer some leeway and flexibility for dealing with intermittent health problems. Respondents are asked to indicate their level of agreement with each statement (e.g., “When I’m not feeling well, I can control the pacing of my work”) on a 7-point scale from “completely disagree” to “completely agree”.

#### Chronic illness checklist

A 16-item checklist of chronic health conditions will provide the type and number of conditions reported by participants [55]. The checklist includes conditions related to musculoskeletal pain, arthritis, headaches, cardiovascular disease, asthma, stomach disorders, mental disorders, diabetes, and handicaps.

### Process evaluation

A process evaluation will be conducted alongside the randomized controlled trial to gain insight into the feasibility of this intervention for more widespread dissemination and to detect any particular study challenges that might be identified from more qualitative assessments. The process evaluation will be based on the RE-AIM framework, which consists of five dimensions: Reach, Efficacy/effectiveness, Adoption, Implementation, and Maintenance [56]. The goals of the process evaluation will be to assess the reach of the program (at both the employer and participant level), to identify any dose–response relationships between levels of participation and outcomes, to evaluate adherence to the study protocol, to assess the satisfaction and experiences of workers and facilitators, and to identify facilitators and barriers for future implementation. Information will be collected by quantitative as well as qualitative methods. In the final follow-up survey, participants will be asked whether they might be willing to participate in an in-depth interview that would include the topics shown above. Since it is not feasible to interview all stakeholders, purposeful sampling will be applied based on relevant characteristics of the target population in combination with the answers to the questionnaire.

### Sample size

The estimated effect size for the primary outcome measure (Work Limitations Questionnaire) is based on the size of effect obtained in a prior intervention trial focusing on workers with depression [38]. In that study, the effect size for various WLQ subscales ranged from 0.51 to 0.87 standard deviation units, suggesting a medium effect size, *f* = 0.25. With a target recruitment of 300 volunteers (60 from each organization), an assumed attrition rate of 20 %, and an alpha level of 0.05, the statistical power to detect a medium effect size (*f* = .25) on the primary outcome measure is 0.96 without consideration of the nested design or the possible need for covariates when making pre-post group comparisons. However, given the possible complication of non-equivalent treatment sites and the inclusion of several covariates in the final group comparison [57,58], a power estimate in the range of 0.80 – 0.85 would be a more conservative estimate based on various scenarios simulated in the PASS 11 Power Analysis & Sample Size Software [59].

### Data collection procedure

After providing informed consent, participants will complete a baseline survey and then be assigned to the intervention or control arms using a block randomization schedule (blocked in groups of 20) maintained privately by the project statistician. Six and 12 months after initial recruitment, participants will be mailed a follow-up survey (or provided access to an on-line survey) to provide for re-assessment of primary and secondary outcome measures. Non-responders will be sent two additional reminders, and all participants will receive a payment of $50 for completing each of the 3 research survey assessments (baseline, 6 months, 12 months).

### Statistical analyses

The primary analytic strategy will be to compare the intervention and control groups on changes in outcome measures at 6- and 12-month follow-up using a multilevel linear mixed model that will take into account the employer and the 3 repeated measurements, and also allow for missing data on either the 6- or 12-month assessment. The two primary outcome measures will be work limitations (total work productivity index from the Work Limitations Questionnaire) and work engagement (total score from the Utrecht Work Engagement Scale). Because of the nesting of participants within 5 different employers, a multilevel analysis will be necessary with employer treated as a fixed factor. This will help to account for any systematic differences in the working populations, job demands, and policies and practices within these organizations. We also anticipate the inclusion of at least 3 individual-level covariates (e.g., age, gender, and number of chronic conditions) in the principal analysis if there are relevant group differences at baseline.

A number of background variables will be assessed to check that the randomization has yielded equivalent comparison groups at baseline. Background variables will include: age, gender, level of education, income, number and type of chronic conditions, blue-collar versus white-collar jobs, job stress, hours of work per week, shift work, number of household dependents, and job and industry tenure.

## Discussion

The present study will evaluate the effectiveness of an employer-supported group intervention program designed to benefit workers with chronic physical health conditions. The intervention is based on principles of pain and illness self-management, and we hypothesize that coaching, education, and skills development in this area will improve worker well-being and reduce functional limitations at work. Innovations of the study include a novel adaptation of self-management principles to the workplace context, the involvement of employers in program sponsorship and enrollment, and the assessment of multiple outcome and process evaluation measures that should provide a basis for further research in this area. Design of the study requires attention to issues of feasibility as well as bias and internal validity.

### Methodological considerations

One feasibility concern that was evident from our preliminary exchanges with employers was the need to maintain the privacy of participating workers. This problem was addressed in the study methodology in several ways. First, a worker will not be required to disclose the nature of his or her health problem in order to qualify for study inclusion or as part of group discussions (however, this information will be collected as part of the confidential research questionnaire). While this may lead to a more heterogeneous sampling, we felt it was more important to preserve the workers’ rights to safeguard personal health information. Second, the intervention itself will be scheduled during lunchtime hours or after work, when there will be no need to coordinate work absences with a regular supervisor. Third, the informed consent and study enrollment process will be conducted through the employers’ Employee Assistance Programs (EAPs) or equivalent institutions that are accustomed to dealing with sensitive employee information and where safeguards are already in place to protect the confidentiality of workers. With these methodological enhancements in place, we expect that workers will be able to volunteer without the risk of workplace stigma or embarrassment.

One methodological dilemma was the choice of an appropriate control group. In a recent review on standard patient self-management programs in 19 randomized controlled trials, the experimental condition showed improved outcomes over care as usual, education leaflets, or waiting-list control groups [28]. While a more rigorous “attention control” condition might provide the best guard against a Hawthorne or similar effect (e.g., the control group attending discussion meetings on another topic), this has not been the standard in studies of self-help interventions, as self-care interventions are not commonly perceived by participants as desirable or comforting in the same vein as massage therapy, supportive psychotherapy, or other hands-on or empathic treatments for pain. Thus, we believe that individuals randomized to the wait-list control arm will be unlikely to experience a high level of dejection and disappointment that would represent a serious bias in the measurement of outcomes at follow-up months later.

While our initial intent was to focus on workers with chronic pain only, we were swayed toward a broader enrollment of workers for the following reasons. First, there is existing evidence that self-management interventions are relevant and effective for a broad range of physical health conditions, not just chronic pain [19,21,22,60]. Second, we observed no problems delivering the 10-hour intervention program to a pilot group of community volunteers with a high level of diagnostic heterogeneity. Third, employers expressed concern that specifying “chronic pain” in the description of the program might discourage workers from participating due to potential stigma and embarrassment. Though announcements and advertisements for the program will specify “chronic or recurrent physical symptoms”, there will be no effort to screen potential candidates based on illness or diagnosis.

Measures were chosen for the study in order to address potential concerns of both workers and their employers. For example, the WLQ provides an opportunity to assess intervention benefits in terms of improved work productivity, but the UWES is more focused on the level of psychological attachment workers feel for their jobs. Similarly, measures of sickness absence, turnover intent, etc. are more directed to employer concerns, while measures of self-efficacy and well-being may be of more importance to workers. The intervention itself also strikes a balance between the need for productivity and the need for worksite wellness. Like all SM interventions, the instructional and participatory elements are based on social-cognitive theory and are designed to boost perceptions of mastery and self-efficacy in the workplace.

Though we developed no definitive criteria for the inclusion of employers in the study, the researchers did consider the issue of worksite readiness. Given the nature of the intervention, it would seem ineffective to provide workers with information about workplace self-management without supportive employer policies and practices that would enable communication and problem solving. Thus, employers with a poor wellness culture or adversarial labor-management relations may not be appropriate to host the study. In actuality, benefits of the group intervention approach might be stronger if partnered with a matching organizational effort to improve practices (e.g., supervisor training, participatory ergonomic approaches, etc.) but this was beyond the scope of the current study. Future studies might adopt a more organizational framework, but privacy issues and organizational status quo represent significant hurdles.

One considerable strength of the study is the recruitment and participation of employees at the workplace. This should generate a more representative sampling of affected workers compared with other forms of recruitment (e.g., through medical clinics or patient lists) or when participation in group meetings requires off-site travel. Also, by conducting the study in actual workplaces, the study should provide useful information about feasibility of implementation as an employer-sponsored health program. The randomized, controlled design of the study also provides a strong basis for investigating effectiveness of the intervention.

### Relevance/impact of results

This study will be relevant for workers with chronic physical health conditions and for all employers. However, this approach may be especially relevant in occupational settings with an aging workforce who face concerns of a large portion of their workforce experiencing chronic conditions, and where some normative level of leeway and decision-making autonomy can be afforded to workers with regard to work style and the organization and prioritization of work tasks. The group intervention strategy in this study may also depend on employer policies and procedures that support worker self-management efforts and have a strong health and wellness culture as a foundation. This study will give some insight into the effectiveness of self-management intervention strategies to reduce disability and improve worker well-being. Results of the study will become available in 2015.

## Competing interests

The authors declare that funding for the project was obtained through intramural research funding of the Liberty Mutual Research Institute for Safety, a division of Liberty Mutual Insurance, Boston, MA.

## Authors’ contributions

WS (principal investigator of the study) participated in intervention development and study design, is responsible for all aspects of methodological rigor and scientific integrity, and drafted the manuscript. EB made substantial contributions to study design methodology in terms of statistical and human subject considerations. GP and MN participated in the design of the study and design of the intervention program. RM participated in the design of the study with respect to feasibility and occupational health concerns and is involved in many aspects of data acquisition and interpretation. TT contributed to the creation of the intervention program and conducted the qualitative work that served as the conceptual basis for the study. All authors participated in manuscript development and agreed to have the final version submitted for publication.

## Pre-publication history

The pre-publication history for this paper can be accessed here:

http://www.biomedcentral.com/1471-2458/14/515/prepub
